# Design and Evaluation of Fusion Approach for Combining Brain and Gaze Inputs for Target Selection

**DOI:** 10.3389/fnins.2016.00454

**Published:** 2016-10-07

**Authors:** Andéol Évain, Ferran Argelaguet, Géry Casiez, Nicolas Roussel, Anatole Lécuyer

**Affiliations:** ^1^Université de Rennes 1Rennes, France; ^2^Inria RennesRennes, France; ^3^Université Lille 1Villeneuve d'Ascq, France; ^4^Inria Lille - Nord EuropeVilleneuve d'Ascq, France

**Keywords:** BCI, gaze tracking, hybrid, multiple input, interaction technique

## Abstract

Gaze-based interfaces and Brain-Computer Interfaces (BCIs) allow for hands-free human–computer interaction. In this paper, we investigate the combination of gaze and BCIs. We propose a novel selection technique for 2D target acquisition based on input fusion. This new approach combines the probabilistic models for each input, in order to better estimate the intent of the user. We evaluated its performance against the existing gaze and brain–computer interaction techniques. Twelve participants took part in our study, in which they had to search and select 2D targets with each of the evaluated techniques. Our fusion-based hybrid interaction technique was found to be more reliable than the previous gaze and BCI hybrid interaction techniques for 10 participants over 12, while being 29% faster on average. However, similarly to what has been observed in hybrid gaze-and-speech interaction, gaze-only interaction technique still provides the best performance. Our results should encourage the use of input fusion, as opposed to sequential interaction, in order to design better hybrid interfaces.

## 1. Introduction

Brain–computer interfaces (BCIs) allow the interaction between a user and a machine by the only means of the cerebral activity. Among the different types of BCIs, the ones based on SSVEP detection (a cerebral pattern of the visual cortex observable in response to a specific stimulation, Quan et al., 2013) have shown high performances for target selection, compared to other BCIs (Chen et al., 2014). As a counterpart, SSVEP-based BCIs require the user's eyes to be externally stimulated by a flickering light in order to use it. Most of the time, SSVEP can be used for target selection when the number of targets is limited.

Another approach that has been widely considered in hands-free interaction is gaze driven interfaces. While controlling a cursor through gaze seems intuitive, it suffers from several limitations. The first limitation is physiological. The precise gaze position oscillates quickly around a center fixation point, making it by essence limited to a precision around 1° of visual angle (Zhu and Yang, 2002; Kammerer et al., 2008). A second limitation is technological. The tracking quality can vary greatly depending on many factors, such as the material, the user, the luminosity, etc. The third limitation lays in interaction techniques. While gaze is relatively intuitive for pointing, it lacks a natural activation command (similar to the click of a computer mouse). The most used technique for selection using gaze tracking is the dwell time. A target is selected when the gaze position stays on top of the target for more than a certain time (the dwell time). This technique results in false positive (i.e., unwanted selections) when the user looks at something he did not want to select. This issue is usually referred to as the “Midas Touch problem” (Velichkovsky et al., 1997).

BCIs and gaze tracking show complementary advantages in the context of hands-free interaction: gaze tracking allows to quickly define a region where potential targets of interest can be selected, while SSVEP is suitable for selecting one target in a small set. Using two measures for related types of inputs should enable a better reliability of the resulting measure, by checking the consistency of both channels. Thus, BCIs could help improve the precision of gaze-based interaction, on top of providing a click command. And yet, there is surprisingly little previous work that tried to combine these two input modalities (Zander et al., 2010).

In this paper, we propose a new approach based on input fusion, designed for improving selection time and accuracy. This approach is illustrated by a fully functional interaction technique that was compared to the state of the art. We show that this fusion method outperforms the previously existing sequential method for BCI and gaze tracking hybrid selection.

The paper is organized as follows, first we present the related work on target selection techniques based on gaze tracking, BCI, and both. Second we detail our new approach for hybrid interaction based on input fusion of gaze tracking and BCIs. Then, we describe a controlled experiment in which we evaluated the proposed technique with two previously existing BCI-gaze techniques. Section 5 presents the results of the experiment followed by its discussion in Section 6. Finally, Section 7 provides the concluding remarks.

## 2. Related work

This section presents the most relevant studies related to the scope of this paper. We focus on target selection tasks, in particular on existing gaze- and SSVEP-based methods for target selection.

### 2.1. Target selection

According to Foley et al. (1984), any interaction task can be decomposed into a small set of basic interaction tasks. Foley proposed six types of interaction tasks for human–computer interaction: *select, position, orient, path, quantify*, and *text*. Depending on the interaction context, other basic interaction tasks have been proposed since then. The *select* interaction task is described as : “The user makes a selection from a set of alternatives” (Foley et al., 1984). This set can be a group of commands, or a “collection of displayed entities that form part of the application information presentation.” In human–computer interaction, selection is often performed with a point-and-click paradigm, generally driven by a computer mouse. The performance of an interaction technique for selection is usually measured by Fitts' law. This law is a descriptive model of human movement. It predicts that the time required to rapidly move to a target area is a function of the ratio between the distance to the target and the width of the target. This model is well suited to measure pointing speed, and has thus been widely used for point-and-click selection method where the “pointing” is critical, while the “clicking” is not.

In the specific context of hands-free interaction, other input devices need to be used. Among them, gaze tracking has shown promising results (Velichkovsky et al., 1997; Zhu and Yang, 2002). Speech recognition or BCIs are other alternatives for hands-free interaction (Gürkök et al., 2011). Hands-free interaction methods can rely on a point-and-click paradigm, but in this specific context, the “clicking” is often as problematic as the “pointing” (Velichkovsky et al., 1997; Zander et al., 2010). Gaze tracking, speech recognition, and BCIs all share the particularity of presenting a relatively high error rate, compared to a keyboard or a mouse, for example.

### 2.2. Gaze-based interaction

In order to improve dwell-based techniques, several methods have been proposed such as the *Fish-eye methods* (Ashmore et al., 2005). *Fish-eye methods* magnify (zoom in) the area around the gaze position, thus decreasing the required selection precision, but without addressing the Midas touch problem. However, the omnipresence of the visual deformation can degrade the exploration of the graphical interface. A potential solution is to zoom in only when potential targets are available (Ashmore et al., 2005; Istance et al., 2008). Another solution relies on designing user interfaces specifically suited for gaze-based selection such as hierarchical menus (Kammerer et al., 2008).

### 2.3. SSVEP-based BCIs

When the human eye is stimulated by a flickering stimulus, a brain response can be observed in the cortical visual areas, under the form of an activity at the frequency of stimulation, as well as the harmonics of this frequency. This response is known as Steady-State Visually Evoked Potential (SSVEP). SSVEP interfaces are frequently used for brain–computer interaction (Legeny et al., 2013; Quan et al., 2013), as SSVEP-based BCIs have a high precision and information transfer rate compared to other BCIs (Wang et al., 2010).

The classical usage of SSVEP-based BCIs is target selection (Quan et al., 2013; Shyu et al., 2013). In order to select a target, the user has to focus on the flickering target she wants to select, each visible target being associated to a stimulation at a different frequency. The SSVEP response is detected in the brain activity of the user through the analysis of the EEG data, and the corresponding target is selected. Most of the time, SSVEP-based interfaces are limited to a small number of targets (commonly three targets), although some attempts were successful at using more targets, in a synchronous context (Wang et al., 2010; Manyakov et al., 2013; Chen et al., 2014).

### 2.4. Gaze and EEG based hybrid interaction

The concept of *Hybrid BCI* was originally introduced in Pfurtscheller et al. (2010) and it was defined as a system “composed of two BCIs, or at least one BCI and another system” that fulfills four criteria : “(i) the device must rely on signals recorded directly from the brain; (ii) there must be at least one recordable brain signal that the user can intentionally modulate to effect goal-directed behavior; (iii) real time processing; and (iv) the user must obtain feedback.”

In the past few years, it has been proposed to combine BCIs with a keyboard (Nijholt and Tan, 2008), a computer mouse (Mercier-Ganady et al., 2013), or a joystick (Leeb et al., 2013). Several types of BCIs can also be used at the same time (Li et al., 2010; Fruitet et al., 2011). In Gürkök et al. (2011), participants can switch at will between a SSVEP-based BCI and a speech recognition system. For a more complete review on hybrid BCIs, the interested reader can refer to Pfurtscheller et al. (2010).

All these contributions can be broadly classified in two categories: sequential or simultaneous processing (Pfurtscheller et al., 2010). Hybrid BCIs based on sequential processing use two or more inputs to accomplish two or more interaction tasks. Each input is then responsible for one task. Hybrid BCIs based on simultaneous processing can fuse several inputs in order to achieve a single interaction task (Müller-Putz et al., 2011).

#### 2.4.1. Gaze and BCI-based hybrid interaction

Although the idea of combining BCI and gaze-tracking has been already proposed, it has been marginally explored. Existing works have mainly focused on P300 (Choi et al., 2013) and motor imagery (Zander et al., 2010) BCIs. Regarding P300 paradigms, Choi et al. (2013) combined gaze tracking with a P300-based BCI for a spelling application. Compared to a P300 speller, the number of accessible characters and the detection accuracy are improved. In contrast, Zander et al. proposed to control a 2D cursor with the gaze, and to emulate a mouse “click” with a motor-imagery based brain switch (Zander et al., 2010). They found that interaction using only gaze tracking was a bit faster, but that BCI-based click is a reasonable alternative to dwell time.

Later, Kos'Myna and Tarpin-Bernard (2013) proposed to use both gaze tracking and SSVEP-based BCI for a selection task in the context of a videogame. The gaze tracking allowed for a first selection task (selecting an object), followed by BCI-based selection for a second task (selecting a transformation to apply to the previously selected object). The findings of this study indicate that selection based only on gaze was faster and more intuitive.

So far, attempts at creating hybrid interfaces using EEG and gaze tracking inputs for target selection have focused on sequential methods, and proposed ways to separate the selection into secondary tasks.

Zander et al. (2010) separates the task (selection attribute) into pointing and clicking, while both (Choi et al., 2013) and Kos'Myna and Tarpin-Bernard (2013) use a two-step selection. In this paper, we propose a novel hybrid interaction technique, that simultaneously fusions information from gaze tracking and SSVEP-based BCI at a low level of abstraction.

## 3. Combining gaze and BCI inputs for target selection

This section details the proposed gaze and SSVEP-based hybrid interaction technique that allows simultaneous processing, as defined in Pfurtscheller et al. (2010). Both inputs are combined at a low level of abstraction for a single selection task. Our hypothesis is that the combination of both inputs will lead to better accuracy and higher speed than previous hybrid methods. We also hypothesized that this hybrid approach can outperform dwell time approaches for high density targets, as gaze-based interaction using dwell time is especially sensible to inter-target distance (Miniotas et al., 2006).

### 3.1. Novel approach for combining brain and gaze inputs: the fusion

#### 3.1.1. Concept

The general idea of our new approach is to combine gaze and EEG inputs at a lower level, in order to build a single, more precise, selection command (see Table 1). When the user wants to select a target, both the gaze position and the cerebral activity will be combined in order to estimate the desired target.

**Table 1:** **Comparison of interaction techniques for gaze and/or BCI-based selection**.

**(A) GAZE-ONLY APPROACH**	
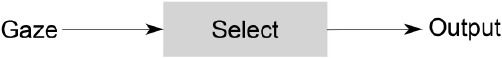	Selection based on gaze only, usually with dwell time (Velichkovsky et al., 1997; Zhu and Yang, 2002; Majaranta and Räihä, 2007; Majaranta et al., 2009)
**(B) HYBRID SEQUENTIAL APPROACH (TASK ATTRIBUTES SEPARATION)**	
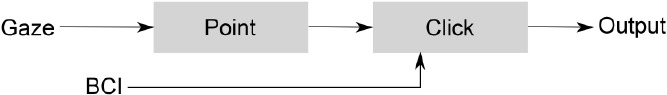	Hybrid interaction with sequential processing: the gaze moves a cursor and the BCI selects the target (Zander et al., 2010)
**(C) HYBRID SEQUENTIAL APPROACH (TWO-STEP SELECTION)**	
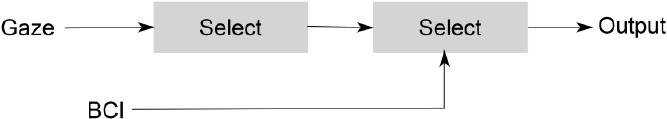	Hybrid interaction with sequential selection. A first selection is done with the gaze only, to determine the set from which a second selection is performed with the BCI only, as in Kos'Myna and Tarpin-Bernard (2013) and Choi et al. (2013).
**(D) HYBRID SIMULTANEOUS APPROACH (OUR APPROACH BASED ON INPUT FUSION)**	
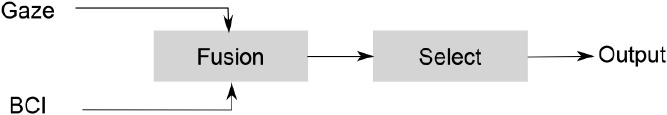	Hybrid interaction based on input fusion. Both inputs are combined to perform a single selection task.

*A, B, C: State-of the art approaches. D: Our novel approach based on input fusion*.

Any interaction system using target selection can be considered as a an estimator of the fact *F*: “the user is trying to select *this* target.” The main idea of the input fusion is to build a probabilistic model of *F*, while taking into account the specificity of the inputs uncertainty. For each input, a model of the distribution of errors is proposed. The resulting models are combined in order to build a higher level estimation of *F*'s likelihood. This information is accumulated over time, until a target is selected when a certainty threshold has been reached. The goal is to allow the user to simply look at any target in order to select it while keeping false positives as low as possible.

#### 3.1.2. Main components

**Figure 1:**
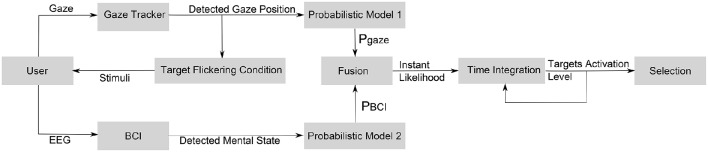
**Schematic diagram of simultaneous processing for the proposed hybrid interaction technique based on the fusion of gaze and EEG inputs**.

The different components of the proposed hybrid interaction technique are depicted in Figure 1. First, the use of SSVEP-based BCI requires a visual stimulus to be associated with each target. In order to overcome the issue of the number of available frequencies of stimulation, a *target flickering condition* is used: only the targets close enough to the detected gaze position flicker.

For both the gaze and the BCI information, a probabilistic model estimates the probability for each target that the user is trying to select (Probabilistic models 1 and 2 on Figure 1). These two simple models of error can be combined, in order to fuse the inputs and build an estimate of the likelihood of *F* at any time. The resulting likelihood is integrated over time in order to reach a satisfying certainty level and to select the target. An activation level is associated to each potential target. For each time step, this activation level is increased by the current likelihood *P*(*F*). Additionally, a decrease *C* of the activation level over time is needed, so that the activation level remains confined. A target is selected when the activation level of this target reaches a pre-defined threshold.

#### 3.1.3. Target flickering condition

In order to define what it means to be “close enough to the detected gaze position,” the threshold of distance is fixed as a function of the standard deviation of the gaze detection accuracy σ. Let us build rules for deciding which target should flicker, by iteratively adding constraint to avoid unwanted behaviors.

- *R*_0_, the rule of supplies limitation: *a maximum of three SSVEP targets can flicker at the same time*.
- *R*_1_, the rule of comparative distance: *Targets closer to the detected gaze position have higher priority*.
- *R*_2_, the “out-of-reach” rule: *Targets do not flicker if their distance to the detected gaze position is higher than* 3 * σ.

The rule of supplies limitation limits the number of targets flickering at the same time, for a better comfort of use. *R*_1_, the rule of comparative distance, seems like a natural way to choose which target should be flickering. However, it seems unnecessary to have a target flicker if it is very far from the gaze, in the case where the target density is low. Hence, the rule *R*_2_ can be added.

Stopping at these rules would be enough to have a well-defined flickering condition, but an issue remains. If more than three targets are closer than the threshold, there would be a phenomenon where the targets that are flickering constantly change, making it extremely difficult for the user to focus on any of them. When two targets are almost equidistant to the gaze position, one might flicker, then the gaze move slightly, and the other takes priority. Then the gaze moves again just by a little, and the first one takes priority again, and so on. In order to avoid this phenomenon, *R*_1_ is replaced by *R*_3_ and *R*_4_:

- *R*_3_, the rule of immediate vicinity: *Targets that are closer than* σ *to the gaze position flickers, except when this rule conflicts with R*_0_, *in which case priority rule R*_1_ *applies*.
- *R*_4_, the rule of preservation: *Targets that were already flickering before will keep flickering, as long as this rule does not conflict with rules R*_0_, *R*_2_*, and R*_3_.

This set of rules provides a flickering condition ensuring that there is never more than three targets flickering at the same time, targets close to the gaze position flickers, and a certain stability of the flickering targets is maintained. Flickering conditions are checked at the gaze tracking refresh rate of 60 Hz.

#### 3.1.4. Gaze precision model

Gaze precision is modeled with the idea that high errors in position detection are unlikely, while minor imprecision is perfectly plausible. Additionally, gaze tracking usually shows a better precision on the horizontal axis than on the vertical one.

Thus, we choose to model gaze precision by *P*_*gaze*_ (see Equations 1 and 2), a two-dimensional Gaussian distribution, centered around the detected gaze position (*x*_*g*_, *y*_*g*_), where the horizontal and vertical variances σ_1_ and σ_2_ can be determined experimentally.

(1) $$\begin{array}{r} N\left(\sigma ,\mu ,x\right)=\frac{1}{\sigma \sqrt{2*\pi }}e^{-\frac{{\left(x-\mu \right)}^{2}}{2\sigma^{2}}} \end{array}$$

(2) $$\begin{array}{r} P_{gaze}\left(x,y\right)=N\left(\sigma_{1},x_{g},x\right)*N\left(\sigma_{2},y_{g},y\right) \end{array}$$

#### 3.1.5. BCI precision model

The BCI precision is modeled by *P*_*BCI*_, a uniform distribution over all targets (*T*_*i*_)_*i*∈{1, …*n*}_, except for the target associated to the stimuli corresponding to the detected frequency *f*, which is given a higher probability *p* (probability that the classifier gives the correct class at any time). *P*_*BCI*_ is *p* for the target chosen by the SSVEP classifier, and the remaining 1 − *p* uniformly distributed across the other targets (see Equation 3).

(3) $$P_{BCI}(T_{i})=\{\begin{array}{cc} p & \text{if }T_{i}\text{ flickers at frequency }f\\ \frac{1-p}{n-1} & \text{otherwise} \end{array}$$

#### 3.1.6. Combining probabilistic models for target selection

The final likelihood estimation is build by combining *P*_*gaze*_ and *P*_*BCI*_:

(4) $$\begin{array}{l} P\left(F\right)=P_{gaze}\left(F\right)*P_{BCI}\left(F\right) \end{array}$$

Finally, the target activation levels evolve as a function of *P*(*F*). At each time step (every 100 ms, following the SSVEP classification output), activation levels are increased by *P*(*F*), and decremented by a constant *C*. This decrease over time ensures that targets can eventually return at rest, it is chosen to be linear in order to keep the number of parameters of the technique small. Formally, at each time step and for each target, the activation level is increased by *P*_BCI_^*^ *P*_*gaze*_ − *C*.

### 3.2. State-of-the-art techniques

#### 3.2.1. Gaze-only and dwell time

The most commonly used approach for hands-free selection relying on gaze tracking remains the dwell time (Majaranta and Räihä, 2007; Majaranta et al., 2009). A target is selected when the gaze is detected to stay on the target for more than the *dwell time*. With the formalism of the fusion method description (see Figure 1), it can be seen as the trivial case with no stimulation nor BCI, and the fusion of inputs is the raw detection of gaze position (see Equations 5 and 6). For any target *T*_*i*_, centered is (*x, y*):

(5) $$P_{gaze}(x,y)=\{\begin{array}{cc} 1 & \text{if }{\left(x_{g}-x_{i}\right)}^{2}+{\left(y_{g}-y_{i}\right)}^{2}<\sigma^{2}\\ 0 & \text{otherwise} \end{array}$$

(6) $$\begin{array}{rcl} P\left(F\right) & = & P_{gaze}\left(F\right) \end{array}$$

The activation level of a target rises at a constant rate when the gaze is detected to be fixed on it, and go back to 0 when the gaze is away. A target is selected when a threshold of activation level (the *dwell time*) is reached.

For this experiment implementation, the detected gaze position is considered to be “on” the target when the distance between the detected gaze position and the center of the target is smaller than σ, the standard deviation of the gaze detection. In other words, the gaze position is treated as a disc of radius σ rather than a point. σ was measured in pre-experiments (see Section 4.2). It is also the radius of the feedback circle. The target activation level decrease linearly in time when the gaze is away, in order to keep the number of parameters small. The rate of decrease was chosen by optimizing the system sensitivity in pre-experiments (see Section 4.5). Refresh rate of the activation level computing was set to 20 Hz.

#### 3.2.2. Hybrid approach based on sequential processing

State-of-the art techniques for hybrid gaze and BCI based target selection (see Section 2.4) use gaze to determine which targets are eligible, and the BCI to trigger the selection (see Table 1). For the sake of comparison, we propose here a reproduction of this approach.

In the formalism used to describe the fusion method (see Figure 1), it can be seen as the case when the gaze is used to determine the target flickering condition, as in the fusion method, but is ignored for the instant likelihood estimation (see Equation 7) The target flickering condition is the same as the one used for the fusion technique (see Section 3.1.3).

(7) $$\begin{array}{r} P\left(F\right)=P_{BCI}\left(F\right) \end{array}$$

If the user is focusing on one flickering stimulation, the SSVEP-based BCI will detect it. As for the fusion method and the dwell time method, an activation level is associated to each target, and is increased by (*P*(*F*) − *C*) at each time step. The rate of decrease *C* was chosen by optimizing the system sensitivity in pre-experiments (see Section 4.5). Activation level is updated at each SSVEP classifier output, leading to a refresh rate of 10 Hz.

### 3.3. Signal processing

In this subsection, we provide the underlining algorithms and methods used in signal processing and classification for both gaze and EEG signals in all three presented interaction techniques. The gaze signal processing is common to all three techniques, while EEG signal processinf is applied for both hybrid methods.

#### 3.3.1. Gaze signal processing

The raw coordinates output given by the gaze tracker are noisy. The resulting trajectory is discontinuous and irregular. In order to get a smooth trajectory, this trajectory is passed through a low-pass exponential filter which rate evolves depending on the variance of the position, as in Casiez et al. (2012). This method allows for rapid shift, while still stabilizing the noisy detection when the gaze is fixed.

#### 3.3.2. EEG signal processing

##### Feature extraction

Features of interest are extracted from the EEG data: the frequency of interest were the three possible frequencies of stimulation: 10, 12, and 15 Hz. A measure of the spectral density of the frequency of interest, as well as of its first harmonic, was computed as in Legeny et al. (2013). The filtered signals for the six channels are processed through two fourth-order common spatial pattern (CSP) filters, in order to optimize the detection of these specific frequencies. The resulting filtered signals are then decomposed in 0.5 s moving windows, with 0.1 s moving steps. If *S*(*f*) is the signal filtered around frequency *f*, the energy spectral density is computed as the average of *S*^2^(*f*) over the time window, and a natural logarithm of this estimated density is computed and used as feature for the following classification algorithm.

##### Classification

Classification is done as in Legeny et al. (2013). A three-class LDA classifier is trained, combining three two-class LDA classifiers, each of them discriminating one class vs. all the others. For each class I of stimulation (defined by its frequency), a two-class LDA classifier is learned, discriminating signals of class I against all the others. This classifier gives *d*_*i*_ an oriented distance to the hyperplane of separation. When used with two classes, such a classifier decides for class I if 0 ≤ *d*_*i*_. In order to combine several two-class LDA classifier to classify between more classes, the chosen class is the one maximizing *d*_*i*_. Previous studies Évain et al. (2016) have found that such a classifier shows a precision of the order of 65% for each 0.5 s time window. A better accuracy can be reached by adding a voting step. In this study, the voting step is included into the interaction technique.

## 4. Experimental evaluation

We performed an experiment evaluating the performances of the three techniques presented in the previous section: two previously existing approaches, and our novel fusion-based method. During this experiment, participants were asked to perform a selection task using the three different techniques. We observed the sensitivity of each method, together with their respective speed of selection.

### 4.1. Participants

Twelve participants took part in our experiment (three women), all right-handed, aged between 22 and 43 (mean = 28, *SD* = 6.2). None of them had vision problems, none of them wore glasses. Two participants (not counted in the 12) were excluded from the experiment because the eye-tracking system was not working properly, these participants were replaced.

Moreover, five additional participants took part in a first pre-experiment in order to assess the gaze tracking accuracy. Finally, 15 additional participants performed a second pre-experiment which aimed at choosing the optimal parameters for each of the three interaction techniques considered in the experiment (five participants each).

### 4.2. Materials and apparatus

Considering that this study presented no risk for either physical or mental health of participants, and that all the participants were healthy adults, this study was exempted from an ethic committees approval. Nevertheless, this study was carried out in accordance with the recommendations of the Declaration of Helsinki. Prior to the experiment, all participants were asked to read and fill a consent form stating their rights and the objective of the experiment. In particular, this consent form recalled that participation was not remunerated, that they had the right to withdraw without prejudice at any moment and without having to give a reason, that all collected data would be anonymized and used exclusively for research purposes.

**Figure 2:**
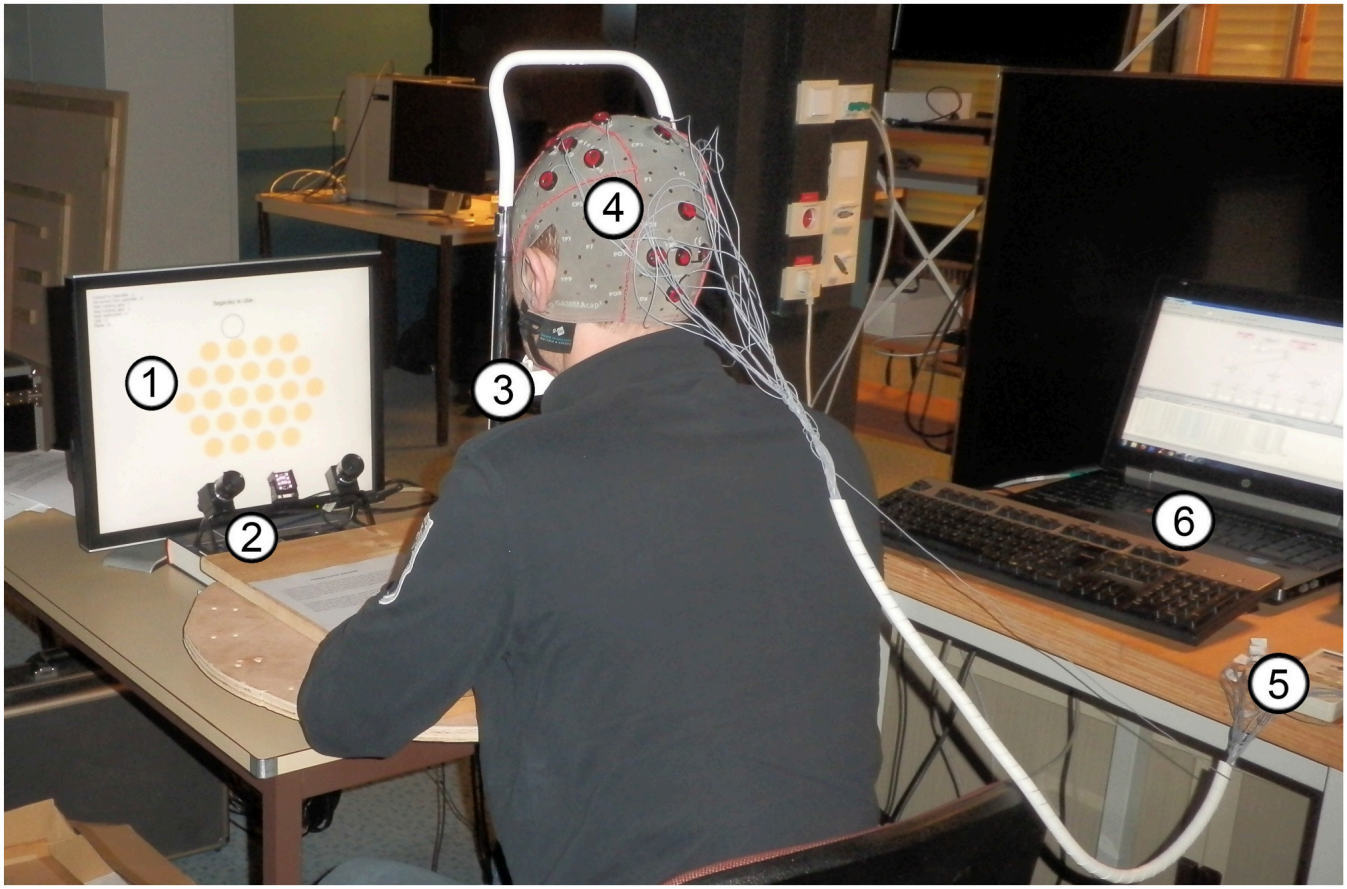
**Experimental setup**. 1, Visual display; 2, Gaze tracker; 3, Chin rest; 4, EEG headset; 5, EEG signal amplifier; 6, Laptop with Openvibe software for EEG signal processing.

Gaze data was measured using a Facelab 5.0 gaze tracker and a chin rest was used to maintain the proper positioning of participant's head (see Figure 2). The variance of the detection on both direction was measured in a pre-experiment on five participants. Gaze tracking was calibrated, and participants were asked to look at a fixed point, while the detected position was recorded. Distance between the eyes and the screen was about 70 cm. We measured a horizontal standard deviation of σ_1_ = 0.78 cm (0.64° of visual angle) and a vertical standard deviation of σ_2_ = 1.49 cm (1.22° of visual angle). Without separating the dimensions, the resulting standard deviation is σ = 1.68 cm. These findings are well in the order of magnitude of the gaze tracker performances^1^.

EEG data was acquired using six electrodes out of a 16-channel system (g.USBAmp, g.tec company, Austria), with a sampling rate of 512 Hz. Electrodes of interest were concentrated above the visual cortex, at position CPz, POz, Oz, Iz, O1, and O2 according to the extended 10–20 system. A reference electrode was located on the right ear, and an additional ground electrode was located on AFz. Channels were amplified and band-pass filtered between 2 and 60 Hz. A notch filter was applied to exclude frequencies between 48 and 52 Hz, corresponding to the power supply frequency band. Electrode impedance was checked to be below 1 kilo-ohm to ensure signal quality. Signal processing was done on OpenViBE running on a dedicated machine (Renard et al., 2010). Visual display was provided by a DELL™Ultrasharp™2007FP 51 cm screen (20.1 inches), with a resolution of 1280 × 1024 pixels, and a refresh rate of 60 Hz.

#### 4.2.1. System calibration

For each participant, the system had to be calibrated. Since BCI calibration takes more time, and that gaze tracking shows various performance depending on the participant, gaze tracking was calibrated first.

For gaze tracking calibration, participants were asked to keep their eyes on a point moving on the screen. Gaze tracking calibration took about 30 s.

BCI was then calibrated. Three SSVEP targets were displayed on the screen. Targets had 3.2 cm of diameter, and were disposed as the corner of an equilateral triangle, 14 cm from each other. Participants were instructed to look at one of them while their brain activity was registered. Targets flickered during 7 s, followed by a 4 s break during which the next target was indicated to the participant. The full BCI calibration took around 3 min. After the BCI calibration, gaze tracking calibration was again checked, in order to ensure that it was still valid. If not, gaze tracking could be re-calibrated.

### 4.3. Task

**Figure 3:**
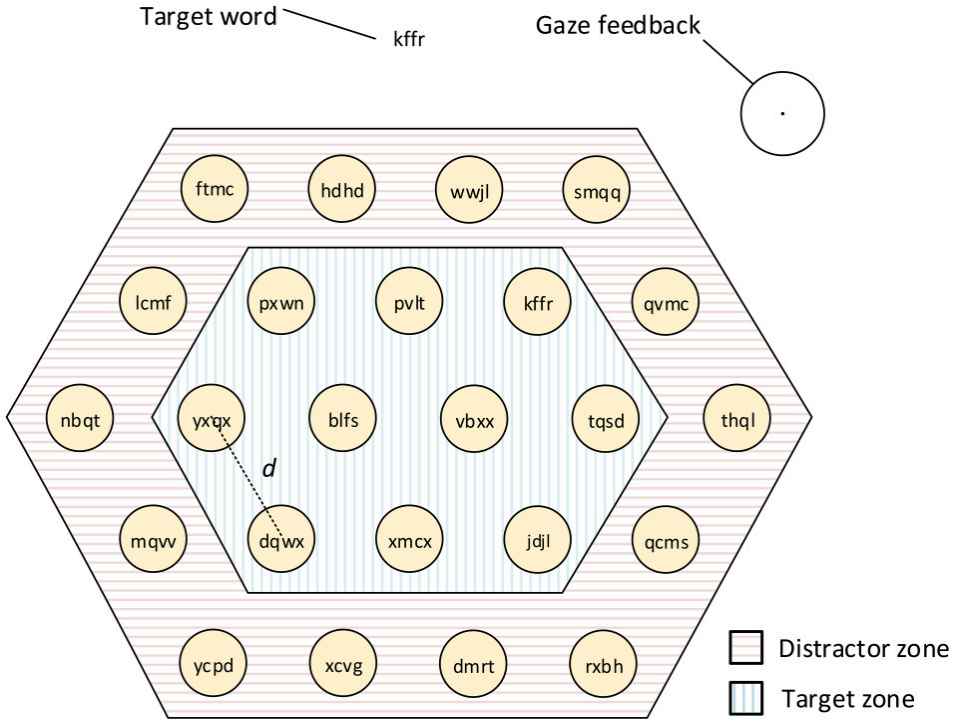
**Design of the experimental task**. The user has to look for the goal word displayed at the top of the screen, then, the user has to select the target with the exact same word. The detected gaze position is displayed under the form of a circle and a central point (visual feedback). For all trials the size of the targets remained constant, and only the length of the target word and the separation (*d*) between targets varied. The targets at the outer circle were distractors in which the target word was never placed.

Once the calibration was done, the core of the experiment could start. For each trial, a *goal* word was displayed at the top of the screen. Several targets were displayed below on an hexagonal layout (see Figure 3). Random words were displayed on each target. These words were generated as in Zander et al. (2010), i.e., they were sequences of random letters uniformly distributed over the consonants. One of the target words matched the goal. Participants' task was to find the target with the *goal*, and to select it. The selection technique varied depending on the block, and was always explained to the participant beforehand.

When a target is close enough to the detected gaze position, a flickering stimulation is superposed on the target (except for the dwell time interaction technique, based on gaze only). Stimuli are circles flickering between black and white at frequency 10, 12, and 15 Hz. A fully opaque stimulation would hide the text behind it, but a good contrast of luminosity is needed for SSVEP detection. Thus, stimulation were given an opacity of 2/3. A maximum of three targets could flicker at the same time. Stimulation size was 3.6 cm of diameter. For a participant seated at 70 cm of the screen, this size correspond to 3° of visual angle. It was found to be a good trade-off between the size of the stimulation and the SSVEP accuracy in NG et al. (2012).

At any time, a feedback indicates the detected gaze position to the user, under the form of a circle. The radius of this circle corresponds to the measured standard deviation of gaze tracking accuracy (1.6 cm). Additionally, the goal target was never on the outer layer, in order to avoid changes in the number of neighbors distractors. Participants were aware of this particularity.

#### 4.3.1. Evaluation criteria

When the error rate is more critical than the pointing time, alternatives to Fitts' law can be used as metrics.

For each selection task, three outcomes are possible :

- True Positive (TP): The participant succeeds in selecting the right target.
- False Positive (FP): The participant accidentally selects a distractor.
- Miss: The participant could not select anything after a time limit of 10 s. This time limit allows to avoid cases where the participant does not manage to select a target, and the trial lasts too long.

The hit-false rate (also called sensitivity, or *d*′) is widely used as a metric of precision (Stillman, 1993; Verde et al., 2006). This measure takes into account TP, FP, and Miss at the same time. *d*′ is a measure of sensitivity defined as *d*′ = (*#TP* − *#FP*)/(*#TP* + *#FP* + *#Miss*). *d*′ is equivalent to counting 1 point for each success, −1 for each error, and 0 when no selection is done (the result being normalized by the number of trials). Most interaction contexts complete the property *A*0: *A false positive leads the user to select an undo command, and thus having one more command to issue before being able to try again*. For any interaction context under *A*0, *d*′ is the expectancy of the number of effective commands issued by trial, as a function of the true positive rate, the false positive rate, and the miss rate. A sensitivity *d*′ lower than 0 indicates that interaction is not possible in practice within assumption *A*0.

### 4.4. Experimental design

Three independent variables were considered in the experiment. First, the interaction technique which had three levels: gaze-only, sequential, fusion. Second, the length of the target word. Words could be either four or seven letters long as in Zander et al. (2010). Finally, the distance between targets, which had three levels: small, medium and long. Since the targets have a diameter of 2.68 cm, the three distances were :

- Short distance : *d*_1_ = 2.68 cm apart. Targets are touching each other. Density is $d_{1}=pi/2\sqrt{3}=90.7\%$.
- Medium distance : $d_{2}=\sqrt{2}*d_{1}=3.79$ cm apart. Density is *d*_2_ = *d*_1_/2 = 45.3%.
- Long distance : $d_{3}=\sqrt{2}*d_{2}=2*d_{1}=5.35$ cm apart. Density is *d*_3_ = *d*_1_/4 = 22.7%.

The experiment was divided into three blocks. For each block, a different interaction technique was used. The order of the interaction techniques was counterbalanced between the participants. For each block, nine trials were performed for each combination of factor, leading to a total of 81 trials per block, performed in a fully randomized order. The full duration of the experiment was about 45 min, including the breaks and calibration time.

The independent variables were the interaction techniques (three techniques), the length of the target word (two length: four and seven letters), and the distance between targets (three levels). Expectations were that longer goal words and short distances would have a negative impact on performances, resulting in more false selections, and longer task completion times. We expected the gaze-only interaction technique to perform better than the hybrid approach based on sequential processing. The fusion method was expected to perform better than the sequential method, and possibly even better than the gaze-only.

### 4.5. Parameters optimization

For each of the interaction techniques considered (see Section 3), a different set of parameters have to be defined. As we could not find optimal configurations in the literature, in order to ensure an optimal configuration we performed a pre-experiment in which we tested several parameter configurations.

optimized parameters were:

- The **selection threshold** *T* describes the activation level at which a target is effectively selected (see Section 3). In the case of the dwell time method, this threshold is simply the dwell time.
- The **decrease rate** *C* describes the rate of linear decrease of the activation level, when the inputs do not point toward this target (parameter *C* for each method in Section 3).

The task was the same as the one described in Section 4.3. The “threshold” and “decrease” parameters varied across the experiment, in order to exploratory search the ones that maximize the sensitivity measure *d*′. For this pre-experiment, the word length for seven letters, and the distance between targets was 3.79 cm.

A dynamic search was used to quickly find the optimal parameters. The goal is to find the pair (*T*_*i*_, *C*_*i*_) that maximizes *d*′. For each participant, tests are performed to compute *d*′ for nine pairs of parameters, combining three values of *T*, and three values of *C*.

- The first participant is tested on values in {*T*_0_/2, *T*_0_, 2*T_0_}*{*C*_0_/2, *C*_0_, 2 * *C*_0_}. The resulting *d*′ are registered.
- For each participant after that, tested values are {*T*_*o*_*pt*/2, *T*_*o*_*pt*, 2 * *T*_*o*_*pt*} * {*C*_*o*_*pt*/2, *C*_*o*_*pt*, 2 * *C*_*o*_*pt*}, with (*T*_*o*_*pt, C*_*o*_*pt*) being the pair of parameters that resulted in the highest *d*′ on average on all the previous participants [(*T*_*o*_*pt, C*_*o*_*pt*) is updated after each participant].

This searching method allows to converge to the best parameters order of magnitude, and refine the evaluation precision for the most likely optimum. Search is stopped when the current value of the pair (*T*_*o*_*pt, C*_*o*_*pt*) is based on at least five participants.

#### 4.5.1. Pre-experiment results

The optimal parameters found during this pre-experiment can be interpreted by the resulting smallest possible time of target activation, and the smallest possible time of deactivation. Selection threshold *T* and decrease rate *C* are parameters unsuited for comparison, as they depend on the technique refresh rate *r*, and on the maximal activation level increment *maxI* achievable at each time step, which depends on the probabilistic model. For the sake of interpretability, we provide the smallest possible activation time, computed as *T*/(*r* * *maxI*), and the smallest possible deactivation time, computed as *T*/(*r* * *C*). A high minimal time of activation, or a small time of deactivation, denotes that the optimization of parameters resulted in a rather conservative approach.

The resulting optimal parameters found in this pre-experiment (see Table 2) were used for the main experiment. Overall, the sequential method is prone to generate false positives, because of the limited precision of the BCI, the raw accuracy of the BCI classifier *p* is estimated at 65%, based on previous studies with similar design and signal processing (Évain et al., 2016). Thus, in order to optimize the sensitivity, the chosen parameters were quite conservative, with a high threshold of activation. Information is integrated over a long time to allow selection. Dwell time was fixed at 1 s, which is consistent with previous research (Ashmore et al., 2005). For our fusion method, both inputs need to be consistent in order to get a significant rise in activation level. The parameters chosen by sensitivity optimization allow a very quick selection when these inputs are consistent. As a compensation to avoid too many false positives, the activation level decreases faster (short memory) than for the sequential method when the inputs are not consistent.

**Table 2:** **Results of pre-experiments and optimization**.

**Method**	**Smallest possible activation time (s)**	**Smallest possible deactivation time (s)**
Gaze-only	1	1
Sequential hybrid	3.33	5
Fusion	0.4	1.3

*Thresholds of activation, and decrease activation level rate for all three interaction techniques. Sequential BCI need to integrate information for a long time before taking an informed decision. At the opposite, the fusion hybrid integration can take a decision quickly if the input are consistent, but activation decreases faster*.

## 5. Data analysis and results

### 5.1. Task performance

**Figure 4:**
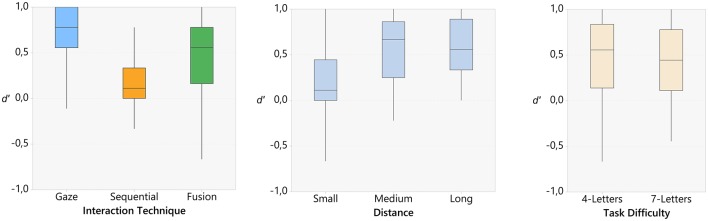
**Boxplots for the sensitivity results, (left) technique, (center) distance, and (right) task difficulty**.

Results of the experiment are displayed in Table 3. We performed a three-way ANOVA considering as factors the interaction technique, the inter-targets distance and the task difficulty vs. the sensitivity (*d*′). All factors being within-subjects (see Figure 4). All three factors were found to have a significant influence on *d*′.

**Table 3:** **Success rates for the three interaction techniques, and the resulting sensitivity (see Appendix for individual results)**.

**Method**	**Correct (%)**	**Miss (%)**	**Error (%)**	**Sensitivity (d′)**
Gaze	82.5	6.8	10.7	0.73
Sequential	20.8	73.7	5.6	0.18
Fusion	55.5	29.9	14.6	0.44

The ANOVA showed a main effect on all three factors: interaction technique [*F*_(2, 22)_ = 34.45, *p* < 0.001, $\eta_{p}^{2}$ < 0.76], inter-targets distance [*F*_(2, 22)_ = 61.13, *p* < 0.001, $\eta_{p}^{2}$ < 0.85], and task difficulty [*F*_(2, 11)_ = 8.19, *p* < 0.05, $\eta_{p}^{2}$ < 0.43]. In addition, we also observed an interaction effect between technique and distance [*F*_(2, 44)_ = 4.00, *p* < 0.01, $\eta_{p}^{2}$ < 0.27].

*Post-hoc* tests (Bonferroni, α < 0.05) showed that the sensitivity for the fusion technique (*M* = 0.44, *SD* = 0.37) was significantly higher than for the sequential technique (*M* = 0.18, *SD* = 0.24), while remaining lower than for the gaze-only technique (*M* = 0.73, *SD* = 0.33).

Regarding inter-targets distance, the close condition (*M* = 0.20, *SD* = 0.36) leaded to the lowest *d*′ [compared to medium: (*M* = 0.56, *SD* = 0.36) and far: (*M* = 0.59, *SD* = 0.32)]. Medium and far conditions were not significantly different. Finally, *d*′ was significantly lower for seven-letters words (*M* = 0.41, *SD* = 0.38) than for four-letters words (*M* = 0.49, *SD* = 0.39). *Post-hoc* tests did not show any conclusive interaction effect between technique and distance.

Additionally, Table 3 provides the breakdown for the trial outcome for each interaction technique. We observe that the decreased performance for the sequential technique is mainly due to the increased number of misses.

### 5.2. Selection time

We conducted a three-way ANOVA analysis of the interaction technique, the inter-targets distance, and the task difficulty vs. the selection time for successful trials. We found a main effect of the interaction technique [*F*_(2, 22)_ = 110.10, *p* < 0.001, $\eta_{p}^{2}$ < 0.91] and the task difficulty [*F*_(2, 11)_ = 7.95, *p* < 0.05, $\eta_{p}^{2}$ < 0.42]. There was no main effect of distance and no significant interaction effects were found.

*Post-hoc* pairwise comparisons revealed that the selection time with the gaze-only technique (*M* = 5.45s, *SD* = 0.85s) was significantly smaller than with the fusion technique (*M* = 6.23s, *SD* = 1.10s), itself smaller than with the sequential technique (*M* = 8.19s, *SD* = 0.95s). In addition four-letter tasks leaded to significantly faster selections (*M* = 6.35s, *SD* = 1.58s) than seven-letter tasks (*M* = 6.72s, *SD* = 1.33s).

#### 5.2.1. Error selection time

The task for each trial can be subdivided in two sub-tasks. First, the user has to search the goal target (search sub-task) and second, the user has to issue the selection trigger (selection sub-task). Selection errors can occur during both of these phases. During the search sub-task, users can involuntarily select one target just by staring too much time on it (e.g., while reading it). These are typical Midas touch errors. In contrast, errors during the second sub-task come from precision limitations, and do not fall within the Midas touch. The user can wrongly select one target while he is trying to select another one.

False positive trials, when a target that is not the correct goal is selected, can be very instructive. In particular, we noticed that most of these errors occurred on targets that are neighbors of the goal. This particularity indicates that most errors occur while the participant has already found the right target, and is trying to select it. We decomposed false positive into two categories:

- Search phase errors: A target has been selected by mistake when the participant was searching for the correct target. This type of error should occur in the first few seconds of the trial, and can be on any target.
- Selection phase errors: A target has been selected by mistake when the participant had already found the correct target, and was trying to select it. Almost all of these errors should be selection of a neighbor target, and they can occur a long time after the trial start.

**Figure 5:**
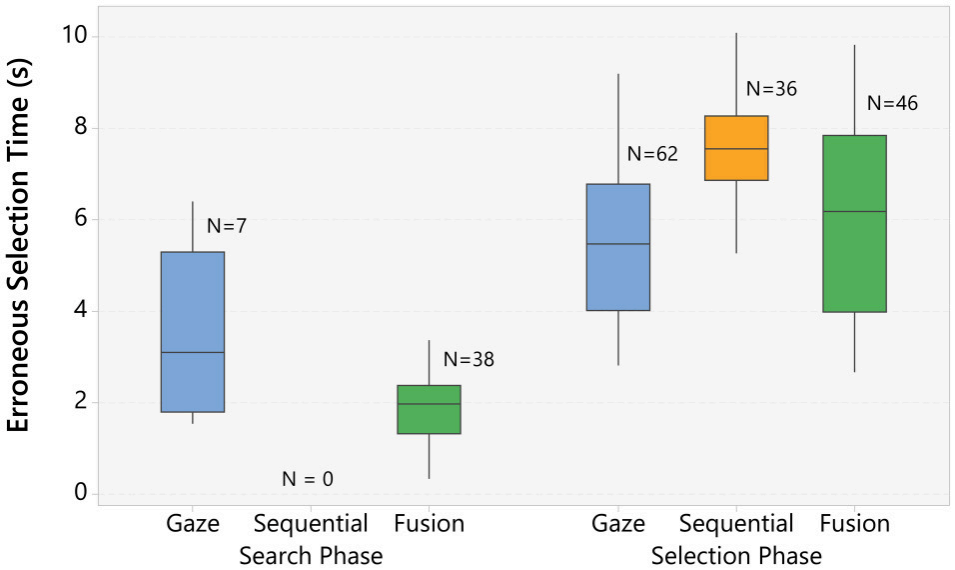
**Boxplot of the mean error selection time grouped by selection technique and whether the selected target was a neighbor or not of the real target**. The number of errors, and the individual values for each error are also provided in the plot.

Figure 5 presents the mean selection time for the erroneous selections. We observe that, for the gaze-only technique, most errors (89%) occur during the selection sub-task. For the sequential technique, all errors resulted in the selection of a target in the neighborhood of the main target and happened after 5 s (see Table 4). This method is very conservative, and prevents early errors. As a counterpart, a lot of trials end up with the time limit. Finally, for the fusion technique, we observe that the number of errors are evenly split between the search and the fusion sub-tasks.

**Table 4:** **Errors analysis**.

**Technique**	**Neighbor errors**	**Non-neighbor errors**
Gaze-only	*M* = 5.58 s, *SD* = 1.59 s, *N* = 59	*M* = 3.33 s, *SD* = 1.53 s, *N* = 10
Sequential	*M* = 7.45 s, *SD* = 1.38 s, *N* = 36	None
Fusion	*M* = 6.12 s, *SD* = 2.06 s, *N* = 44	*M* = 1.97 s, *SD* = 0.98 s, *N* = 40

*means and standard deviations of error timing according to the relationship between the correct target and the selected target*.

### 5.3. Questionnaires results

Participants were asked to rate the fatigue, mental effort, effectiveness, feeling of control, and visual comfort associated to each technique using a seven-point Likert scale. We performed a Friedman test on the rating of fatigue, mental effort, effectiveness, control, and visual comfort, at *p* < 0.05 level. All criteria were significantly influenced by the interaction technique.

*Post-hoc* pairwise comparisons (Wilcoxon) revealed that that fatigue was higher with the sequential method than with the gaze-only or fusion methods (both *p* < 0.05). No significant differences appeared between gaze-only and fusion methods. Mental effort was judged to be lower for the gaze-only method than for the sequential and fusion methods (all *p* < 0.01). No significant differences appeared between sequential and fusion methods. Effectiveness was rated higher for the gaze-only method, followed by fusion, and then sequential. All effects were significant (*p* < 0.05). Control was rated higher for the gaze-only method, followed by fusion, and then sequential. All effects were significant (*p* < 0.05). Finally, visual comfort was rated higher for the gaze-only method, followed by fusion, and then sequential. All effects were significant (*p* < 0.05).

Furthermore, participants were also asked to rank the interaction techniques by preference. All participants preferred our fusion technique over the sequential technique. Eleven of them ordered the techniques as *gaze*−*only* > *fusion* > *sequential*, while one chose *fusion* > *gaze*−*only* > *sequential*. Overall, participants showed a strong preference related to the performance of each method. Namely, they preferred the gaze-only, followed by the fusion and then the sequential technique. Open comments indicated that this choice is influenced both by the accuracy and speed, and because the flickering stimulation is judged to be tiring by the participants.

### 5.4. Results summary

Our fusion technique was found to be faster and less prone to error than the sequential hybrid technique. This results in a higher sensitivity. Participants also preferred it. Overall, the gaze-only method had still higher speed and accuracy than the two hybrid methods.

Task complexity (length of words) is of little influence on the selection accuracy, but does influence the selection time. This result is similar to what was observed in Zander et al. (2010). Overall, a short distance between targets leads to lower *d*′, but does not seem to influence the selection time. Additionally, we observed that distractors close to the targets are more likely to be selected by mistake.

## 6. Discussion

The novel approach that we proposed, based on input fusion, was found to be faster and more reliable than the previously existing approach, based on sequential input processing. This observation is present for all tested level of target density and task complexity. This demonstrates the feasibility of hybrid interfaces using gaze detection and SSVEP-based BCI simultaneously for a single task.

Several factors can influence the accuracy and speed of selection. We observed that a close distance between targets leads to more selection errors. This effect was expected, as both gaze tracker and SSVEP-based BCIs are known to be sensitive to distance between targets. The difficulty of the search task, manipulated by the length of the *goal* word, was found to influence the selection time. This effect had already been observed in Zander et al. (2010). However, *goal* complexity seems to have little influence on the system sensitivity, meaning that a high complexity task requires more time, but has a similar end result.

The user task considered in this study can be decomposed into two phases. First, participants look for the *goal* target. During this phase, they may accidentally select unwanted targets (Midas Touch). When this first phase is over, users try to select a desired and well identified target. During this second phase, they may accidentally select another target. Typically a neighbor of the desired one, especially if the distance between targets is small. This problem is closely related to the gaze tracking accuracy limitation. We could observe that in practice, most errors occur during the second phase. This finding modulates the importance of the factors of interest. In particular, *goal* complexity influences the completion time of the first phase. We believe that this is why *goal* complexity has a strong influence on the overall task complexion time, but not on the system sensitivity. At the opposite, targets density does not influence the search time, but is a critical factor for errors of selection during the second phase.

For hands-free interaction techniques for selection, a trade-off usually has to be made between the difficulty of the selection and its accuracy. While a conservative interaction technique avoids unwanted selections, it increases the difficulty to select the desired target. At the opposite, other choices of interaction parameters may lead to easy selection, but can also be responsible of a lot of unwanted selections. Interaction parameters need to be set carefully. In this study, we chose to set these parameters by optimizing a sensitivity measure *d*′, focusing on task completion. This optimization ensures a balance between speed and robustness, and allows a fair comparison between interaction techniques. We observed that the sequential hybrid technique is best suited to gather information over a long period, and take action only when enough information is gathered. By contrast, the fusion-based hybrid selection takes very quick decision when the inputs are consistent, and forgets quickly about old data if inputs stop pointing toward selection.

Finally, the dwell time method was found to be more efficient for all criteria. Interestingly, a study comparing interaction based on gaze only with a hybrid interaction based on both gaze and speech recognition found similar results: “Contrary to our expectations, an input device solely based on eye gazes turned out to be superior to the combined gaze- and speech-based device” (Kammerer et al., 2008). In order to efficiently use a BCI input, we believe that the classification accuracy needs to be improved. With the current level of BCI reliability, a hybrid interaction method would need to give a very low weight to the BCI input, and to favor the gaze tracking. However, even if a gain in accuracy can be obtained, the discomfort caused by the flickering might overcome the benefit.

A possible trail for future improvements of hands-free selection techniques could be the use of SSVEP-based BCIs allowing a selection among a high number of targets, similar to Wang et al. (2010), Manyakov et al. (2013), and Chen et al. (2014). User comfort when using such systems needs to be addressed. Additionally, these systems have yet to be tested in a self-paced context. The challenge of limiting false positives remains open. One idea could be to use gaze tracking as a “double-check” modality, in order to correct SSVEP false positives. Considering our results in this study, we would advise the use of a fusion-based approach instead.

Traditionally, BCIs can be separated in three categories : active BCIs, when the user is actively trying to use the BCI as an input for interaction, without external stimulation (e.g., motor imagery-based BCIs). Reactive BCIs, when the user receives external stimulation, and interact by focusing his attention on a specific stimulation (e.g., P300 and SSVEP-based BCIs), and passive BCIs, when the user does not need to consciously use the BCI. Instead, the BCI measures the user mental state, and monitors the interaction accordingly. For hands-free selection, these three approaches have pros and cons : Active BCIs require training from the user, and lack accuracy. Reactive BCIs can be uncomfortable, and, as this study shows, require improvements before becoming a useful addition to gaze-based interaction. Finally, passive BCIs could be used together with gaze tracking, in an hybrid interaction setting, but their potential contribution is still to be explored.

## 7. Conclusion

We proposed a new approach for hybrid brain-and-gaze interfaces, based on the fusion of inputs. We found that this method was faster and more accurate than the previously existing hybrid methods based on sequential processing. However, this improved speed and accuracy remains lower than those of interaction based on gaze only. In order to outperform the methods based on gaze only, future hybrid interfaces for target selection could be based on similar fusion approach, rather than on sequential selection methods. In particular, progresses in signal processing can be directly included within our model.

## Author contributions

All authors designed the study. AÉ developed the experimental program and performed the experiment. AÉ and FA conducted the analyses and wrote the manuscript. All authors contributed to the interpretation of the results. AL, GC, and NR supervised the research and revised the manuscript. All the authors read and approved the final manuscript.

### Conflict of interest statement

The authors declare that the research was conducted in the absence of any commercial or financial relationships that could be construed as a potential conflict of interest.

## Footnotes

^1^http://www.ekstremmakina.com/EKSTREM/product/facelab/index.html.

## Appendix

**Table A1:** **Individual results for hit, miss, and false positive rates, along with the resulting sensitivity *d*′**.

**Subject**	**Method**	**Hit**	**Miss**	**FP**	***d*′**
Subject 1	Gaze-only	0.8	0.09	0.11	0.69
	Sequential	0.33	0.65	0.02	0.31
	Fusion	0.41	0.43	0.17	0.24
Subject 2	Gaze-only	0.81	0.04	0.15	0.67
	Sequential	0.13	0.83	0.04	0.09
	Fusion	0.69	0.28	0.04	0.65
Subject 3	Gaze-only	0.69	0.19	0.13	0.56
	Sequential	0.19	0.78	0.04	0.15
	Fusion	0.59	0.31	0.09	0.50
Subject 4	Gaze-only	0.91	0.02	0.07	0.83
	Sequential	0.28	0.70	0.02	0.26
	Fusion	0.80	0.11	0.09	0.70
Subject 5	Gaze-only	0.59	0.24	0.17	0.43
	Sequential	0.14	0.78	0.08	0.06
	Fusion	0.17	0.46	0.37	−0.20
Subject 6	Gaze-only	0.89	0.00	0.11	0.78
	Sequential	0.06	0.89	0.06	0.00
	Fusion	0.63	0.30	0.07	0.56
Subject 7	Gaze-only	0.91	0.02	0.07	0.83
	Sequential	0.24	0.67	0.09	0.15
	Fusion	0.52	0.33	0.15	0.37
Subject 8	Gaze-only	0.89	0.02	0.09	0.80
	Sequential	0.17	0.78	0.06	0.11
	Fusion	0.73	0.18	0.10	0.63
Subject 9	Gaze-only	0.94	0.00	0.06	0.89
	Sequential	0.35	0.59	0.06	0.30
	Fusion	0.52	0.26	0.22	0.30
Subject 10	Gaze-only	0.87	0.02	0.11	0.76
	Sequential	0.31	0.65	0.04	0.28
	Fusion	0.46	0.41	0.13	0.33
Subject 11	Gaze-only	0.81	0.06	0.13	0.69
	Sequential	0.48	0.52	0.00	0.48
	Fusion	0.65	0.26	0.09	0.56
Subject 12	Gaze-only	0.91	0.02	0.07	0.83
	Sequential	0.20	0.67	0.13	0.07
	Fusion	0.74	0.20	0.06	0.69
